# Ceratosaur palaeobiology: new insights on evolution and ecology of the southern rulers

**DOI:** 10.1038/s41598-018-28154-x

**Published:** 2018-06-27

**Authors:** Rafael Delcourt

**Affiliations:** 1 Universidade Estadual de Campinas (UNICAMP), Instituto de Geociências, Rua Carlos Gomes, 250, 13083-855 Campinas, SP Brazil; 2 Museu Nacional/Universidade Federal do Rio de Janeiro, Departamento de Geologia e Paleontologia, 20940-040 Rio de Janeiro, RJ Brazil; 3 Department of Zoology, Trinity College Dublin, Dublin, 2 Ireland

**Keywords:** Palaeontology, Palaeontology, Palaeontology, Palaeontology, Ecology

## Abstract

Ceratosaur theropods ruled the Southern Hemisphere until the end of the Late Cretaceous. However, their origin was earlier, during the Early Jurassic, a fact which allowed the group to reach great morphological diversity. The body plans of the two main branches (Noasauridae and new name Etrigansauria: Ceratosauridae + Abelisauridae) are quite different; nevertheless, they are sister taxa. Abelisaurids have lost the ability to grasp in the most derived taxa, but the reduced forelimb might have had some display function. The ontogenetic changes are well known in *Limusaurus* which lost all their teeth and probably changed the dietary preference at maturity. The results presented here suggest that abelisaurids had different soft tissues on the skull. These tissues might have been associated with evolution of a strong cervicocephalic complex and should have allowed derived taxa (e.g. *Majungasaurus* and *Carnotaurus*) to have low-displacement headbutting matches. The ability to live in different semi-arid environment plus high morphological disparity allowed the ceratosaurs to become an evolutionary success.

## Introduction

Ceratosaurs are theropod dinosaurs known for having extremely reduced forearms and short/deep skulls^1^. Although they are not as famous as their distant relatives, the archetypal tyrannosaurs^2^, the ceratosaurs were abundant and well spread out chronospatially through the Mesozoic^3^ being ecologically important especially in the Southern Hemisphere where most of their remains have been unearthed^4,5^. As the ceratosaurs were the dominant carnivorous dinosaurs of the southern continents, in diversity and ecology during the Late Cretaceous^3,4^, they can be considered the tyrannosaurs’ counterpart. However, research on ceratosaurs has not received the same attention from non-scientific society and they remain mysterious to the lay public.

The type species of the group, *Ceratosaurus nasicornis*, was described in 1884 from a skull and partial postcranial skeleton of the Jurassic of USA^6^, but the clade became better understood with *Carnotaurus sastrei*^7^ which has been subject of several palaeobiological studies^8–11^. In the last three decades the discovery of many species has increased our knowledge of ceratosaurs’ phylogeny^3,12–15^, morphology^1,12,14,16,17^, biogeography^4^, development^1,14,18^ and behaviour^8,9^. These studies have shed new light on the Gondwanan tyrants and allowed for an improved understanding of the evolution and life of theropod dinosaurs.

Here I assess the current state of ceratosaur research, focusing on the origin, phylogenetic relationships and biology of this group in Mesozoic ecosystems. Furthermore, I present new information on soft tissue of abelisaurids bringing additional inference of the behaviour and the use of these tissues. Taxonomic comments are made to clarify and interpret the relationships and nomenclatural issues among the taxa.

## Results and Discussion

### Phylogenetic relationships

Ceratosauria traditionally consists of *Ceratosaurus* and all taxa closer to it than to *Neornithes*^19^. However, taxonomy within Ceratosauria has been complicated. Abelisaurs were formally known as Abelisauroidea (=Ceratosauroidea), that comprises *Carnotaurus*, *Noasaurus* and all their most recent common ancestors and all descendants (see below for further discussion). Ceratosauroidea are included in the clade called Averostra which comprises the taxa related to Ceratosauria and all derived theropods^20^. Approximately 32 Ceratosauroidea genera are currently known with most of the taxa originating from the Late Cretaceous (Table S1).

Ceratosauroidea is traditionally divided into two main branches: the Noasauridae and the Abelisauridae^21^ (but see also the last paragraph for new definitions). This classification has been followed in the recent phylogenetic analyses which have revealed more resolution of the relationships within the clades^3,5,14,15,22^. The relationships between of the two large groups is still being debated; however there are new hypotheses of relationships amongst the noasaurids improving the resolution within the family^14^. In the case of abelisaurids, two main branches divide the South American (called Brachyrostra)^22^ from the European/Indian/Madagascan taxa (previously called Majungasaurinae)^5^. Recent phylogenetic analyses recovered a new clade included in Brachyrostra that comprises the Santonian-Maastrichtian abelisaurids from South America: the Furileusaura^13,15,23^. Nevertheless the relationships amongst furileusaurians are still debated^13^.

The recent analyses of Wang *et al*.^14^ expanded the matrix for phylogenetic relationships of ceratosaurs (744 characters) with dense taxon sampling (198 taxa) including a broad outgroup which better allow to polarize homology statements at the node Ceratosauria. The new hypothesis of Wang *et al*.^14^ suggests *Elaphrosaurus bambergi* and *Limusaurus inextricabilis* as sister taxa as recovered by Rauhut and Carrano^24^. However, in a novel result Wang *et al*.^14^ find that *Berberosaurus* basal within Abelisauridae (=new Etrigansauria, see below), and Ceratosauridae is now composed of *Eoabelisaurus* plus *Ceratosaurus* and *Genyodectes serus*. According to Wang *et al*.^14^, *Ceratosaurus* is united within non-noasaurid ceratosauroids by the following features: (1) fusion of the quadratojugal and quadrate; (2) posterior extent of the posteroventral process of the dentary directly ventral to the posterodorsal process; (3) parapophyses distinctly below the level of the diapophyses in posterior dorsal vertebrae; (4) contact of the pubis and ischial obturator process and (5) transverse infrapopliteal ridge between the medial and lateral femoral condyles. Additionally, *Dahalokely tokana* is recovered as a majungasaurini instead of within Noasauridae as proposed by Farke and Sertich^25^ and Tortosa *et al*.^5^ or within Brachyrostra as suggested by Delcourt^13^ and Filippi *et al*.^15^. This new hypothesis suggests that the origin of ceratosauroids and its two main branches are older than previously thought, with and African origin, decreasing the length of previous ghost linages.

Nevertheless, the inclusion of Ceratosauridae in Abelisauridae as proposed by Wang *et al*.^14^ has important taxonomic implications and some clade definitions must to be done. According to the International Code of Zoological Nomenclature (ICZN)^26^, the family name Ceratosauridae has priority over Abelisauridae because the first was coined in 1884 by Marsh^6^ and the second was coined in 1985 by Bonaparte and Novas^27^. Additionally, according to the Principle of Coordination of ICZN^26^ “a name established for a taxon at any rank in the family group is deemed to have been simultaneously established for nominal taxa at all other ranks in the family group”. It means that once Ceratosauridae is nested in Abelisauroidea, the superfamily Ceratosauroidea is the synonym senior to Abelisauroidea and the synonym junior must be replaced. The definition of Ceratosauroidea here follows the suggestion of Wilson *et al*.^28^ for Abelisauroidea: the clade is composed by *Carnotaurus*, *Noasaurus* and all their most recent common ancestors and all descendants (also including Ceratosauridae). If the phylogenetic hypothesis of Wang *et al*.^14^ is correct, I propose a new clade to include Ceratosauridae and Abelisauridae as well as new definitions for these two families (Table 1):

**Table 1:** The proposed set of definitions for the ceratosaurian clades.

Clade	Definition
Etrigansauria (new clade)	the most inclusive clade containing *Carnotaurus sastrei* and *Ceratosaurus nasicornis* but not *Noasaurus leali*. Etrigansauria means “daemon lizard Etrigan”, a daemon from DC Comics mythology.
Ceratosauridae	the most inclusive clade containing *Ceratosaurus nasicornis* but not *Carnotaurus sastrei*.
Abelisauridae	the most inclusive clade containing *Carnotaurus sastrei* but not *Ceratosaurus nasicornis*.

**Figure 1:**
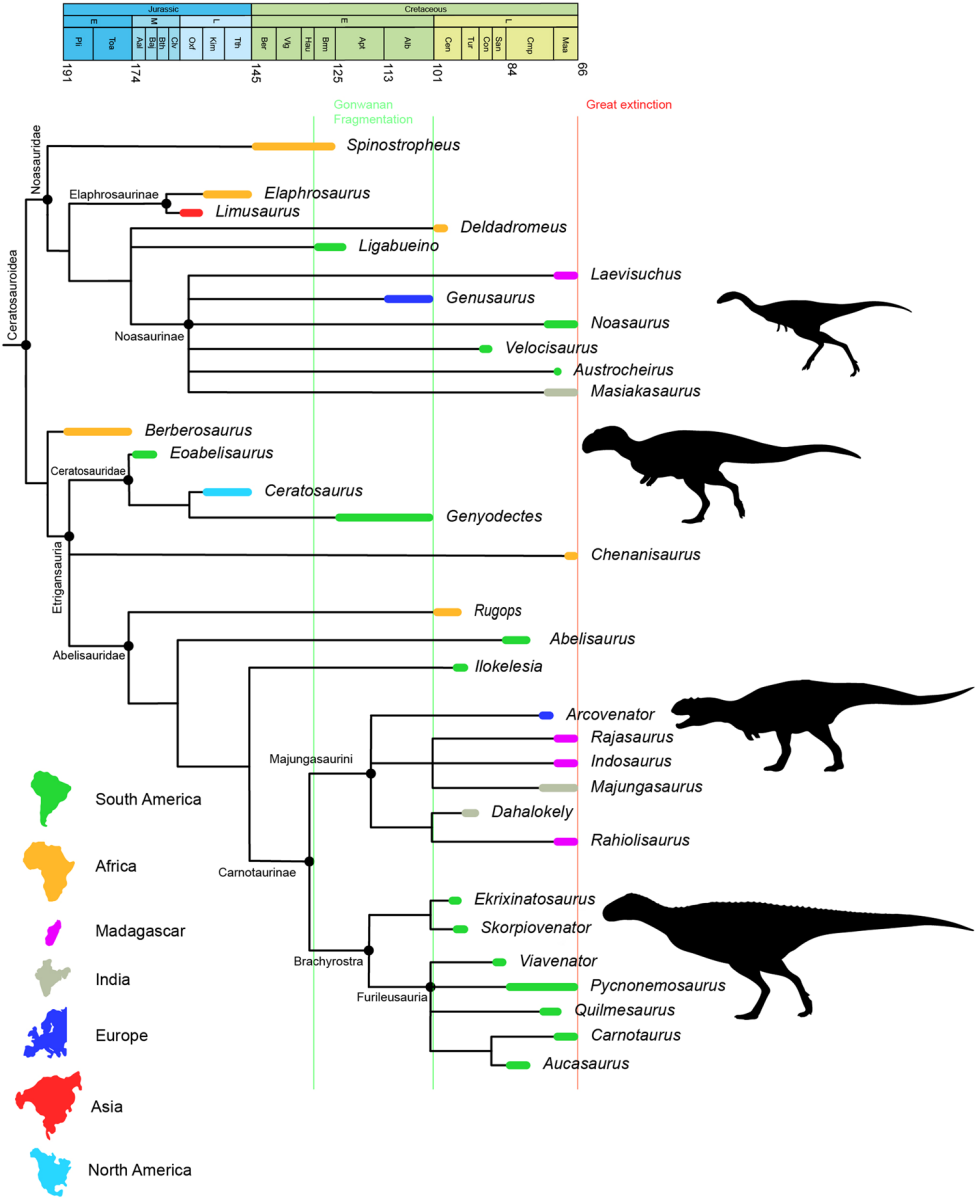
The hypothetical phylogenetic relationships of ceratosaurs based on current topologies. The main source is from Wang *et al*.^15^. The phylogenetic position of *Chenanisaurus* is from Longrich *et al*.^24^ and the *Ligabueino*, *Austrocheirus*, Majungasaurinae and Brachyrostra are from Filippi *et al*.^16^.

Also, it is worth noting that the subfamily Majungasaurinae^5^ in the topology of Wang *et al*.^14^ should be considered a tribe and called Majungasaurini because is inserted in the subfamily Carnotaurinae. This taxonomic change helps to clarify the relationships among Ceratosauroidea and satisfies the nomenclature requirements (Fig. 1). Therefore, in the present contribution I will follow the Wang *et al*.^14^ phylogenetic results. All the phylogenetic definitions used here are in the Supplementary Materials.

The origin of Ceratosauroidea is subject of debate concerning the time of the basal-most taxa. Although its origin has been recovered to the Early/Late Cretaceous (Aptian/Cenomanian, between 126–93.9 My)^3,5,15,25^, some authors suggest it could be earlier, originating in the Early Pliensbachian/Toarcian, between 191–174 My (Early Jurassic)^14,29^ or Aalenian/Bajacian (Middle Jurassic)^12^. These differences hinge on the position of *Berberosaurus liassicus* (Pliensbachian/Toarcian), a ceratosaurian from Morocco known by a partial postcranial skeleton^29^ and the position of *Eoabelisaurus mefi* (Aalenian/Bajacian) a medium-sized etrigansaurian from Argentina known by an almost complete skeleton^12^. Depending on the position of these taxa, the origin of Ceratosauroidea is younger or older. In some analyses, *Berberosaurus* is considered as a basal ceratosaurian^12,13^, a neoceratosaurian^5^, a basal abelisauroid^29^ or sister-taxa of cornisauria^14^. The topology of *Eoabelisaurus* is also controversial, falling out as basal within Ceratosauroidea^5,13^, Abelisauridae^12^ or within Ceratosauridae^14^.

### Ceratosaur anatomy

**Figure 2:**
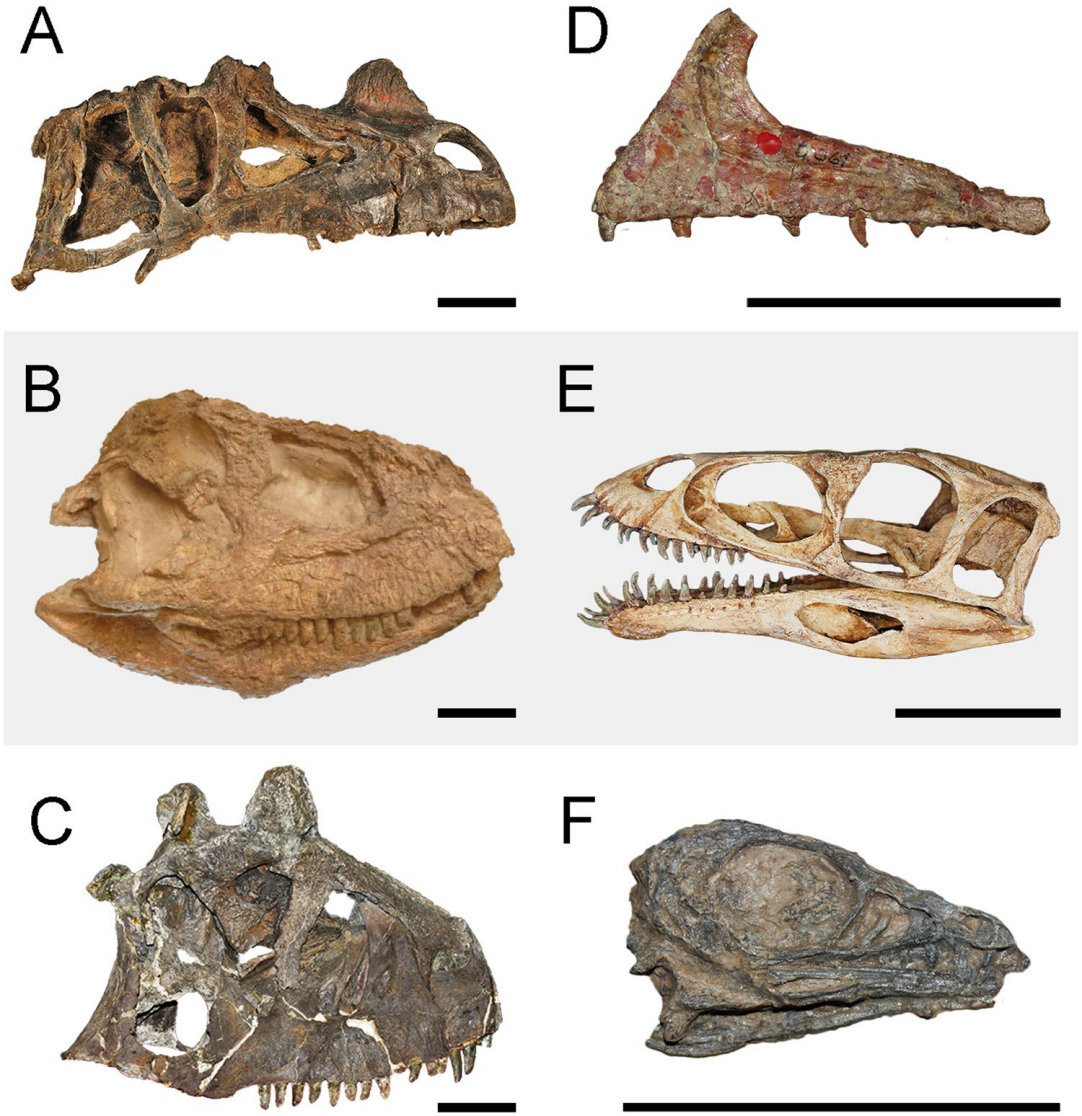
The anatomy of ceratosaurs, showing the variety of cranial morphology in the group. Right lateral side of the skulls of (**A**) *Ceratosaurus* (USNM 4735), (**B**) *Skorpiovenator* (MMCH-PV 48) and (**C**) *Carnotaurus* (MACN-CH 894) (scale bar: 10 cm). Left maxilla of (**D**) *Noasaurus* (PVL 4061; Fundación Miguel Lillo, Tucumán, Argentina); reconstruction of the skull of (**E**) *Masiakasaurus* and left lateral side of the skull of (**F**) *Limusaurus* (IVPP 20093 V; Institute of Vertebrate Paleontology and Paleoanthropology, Beijin, China) (scales bar: 5 cm).

Ceratosauroidea probably has most disparity (morphological variety) of any major theropod group^30^. They could be omnivorous/herbivorous such as in *Limusaurus*^14^, have horns as in *Ceratosaurus*, *Carnotaurus* and *Majungasaurus crenatissimus* or have extreme reduced forelimbs as in *Majungasaurus*, *Aucasaurus garridoi* and *Carnotaurus*^31^. However, the body plans of the main branches (Noasauridae and Etrigansauria) remain respectively similar within each group (Fig. 2).

**Figure 3:**
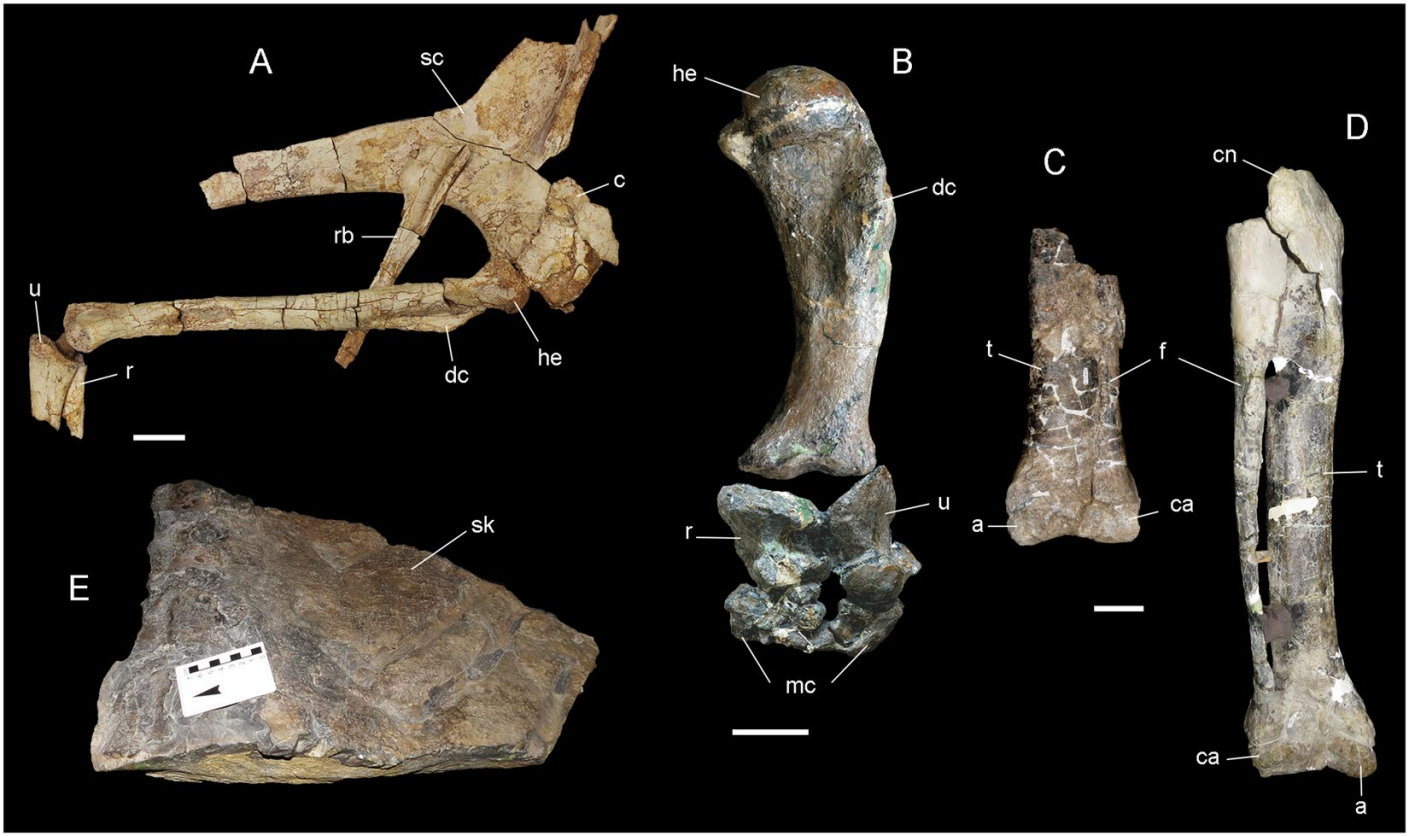
Limbs elements and skin impression of ceratosaurs. (**A**) Pectoral and forelimb of *Deltadromeus* (SGM-Din 2; Ministère de l'Énergie et des Mines, Rabat, Morocco); (**B**) forelimb of *Carnotaurus* (MACN-CH 894); (**C**) distal articulated tibia, fibula, astragalus and calcaneum of *Eoabelisaurus* (MPEF-Pv 3990; Museo Paleontológico ‘Egidio Feruglio’, Trelew, Argentina); (**D**) articulated tibia, fibula, astragalus and calcaneum of *Xenotarsosaurus* (UNPSJB PV 194/1; Universidad Nacional de la Patagonia ‘San Juan Bosco’, Chubut, Argentina) and (**E**) caudal skin impression of *Carnotaurus* (MACN-CH 894). Scale bar: 5 cm. Abbreviations: a, astragalus; c, coracoid; ca, calcaneum; cn, cnemial crest; dc, deltopectoral crest; f, fibula; he, humeral head; mc, metacarpals; r, radio; rb, rib; sc, scapula; sk, skin impression; t, tibia; u, ulna.

Noasaurids tends to be smaller and more gracile than etrigansaurians^1^ with a long neck, small heads, and larger forearms^32–34^. Although the morphology of noasaurids differs substantially from those etrigansaurians, the ilium of Noasaurinae (subfamily included in Noasauridae) is as low as in Carnotaurinae (subfamily included in Abelisauridae) despite the fact that these two groups are not closely related. The skull of noasaurids are long and low compared to those of abelisaurids^17,33^. Interestingly, even among noasaurids the morphology of the skull varies substantially. The skull of *Limusaurus* becomes toothless through ontogeny, likely to meet a change in diet (see below)^14^, whereas the skull of *Masiakasaurus knopfleri* presents strong procumbent dentitions which probably indicate additional divergence from the typical theropod diet^35^. The forearms of noasaurids are poorly known, but as in other ceratosauroids the humerus, radius and ulna are more reduced distally than proximally suggesting that the reduction may have occurred in a modular fashion, from the distal to proximal across the phylogeny^12^. However, the humeri of noasaurids are slenderer than those of abelisaurids (Fig. 3A).

The body plan of etrigansaurians strongly differs from other theropods, and their morphology is more thoroughly known than that of noasaurids^1,4^. Whereas the noasaurids have long skulls, the etrigansaurians have strong and deep skulls, especially those of Brachyrostra which also showed encroachment of the postorbital into the orbit, just beneath the eye^22^. The skull of abelisaurids became shorter and more rugose in more derived taxa. *Ceratosaurus*, *Eoabelisaurus*, and possibly *Genyodectes* have longer skulls compared to those of abelisaurids. The skull’s shortening and deepening started in abelisaurid basal forms, such as the Aptian-Albian *Kryptops palaios* and the Cenomanian *Rugops primus*, both from Niger^36,37^, and reached its extremity in the Carnotaurinae taxa. The skull of *Carnotaurus* is exaggeratedly short and deep compared with those other taxa of the same clade. The skull of *Abelisaurus* was largely reconstructed in the snout as well as in the posterior area^1,3,38^, and taphonomic distortion has modified the proportions and several contacts between elements are missing such as the jugal articulations^3,38^. Therefore, as previously suggested^38^, *Abelisaurus* should have had a shorter skull than was previously reconstructed and frequently reproduced resembling those of Carnotaurinae (e.g. *Majungasaurus*) instead of *Ceratosaurus* (as suggested by Bonaparte and Novas^27^).

Regarding the basal abelisaurids, *Kryptops* was diagnosed based on a left maxilla, several partial vertebrae and ribs and an articulated pelvic girdle and sacrum^36^. However, as noted by Novas *et al*.^4^ and Carrano *et al*.^39^, the pelvic gridle and sacrum of *Kryptops* were found “eroded and free of the rock some 15 meters distant” and have more shared features with tetanurans than abelisaurids. The vertebral non-sacral remains also share features with ceratosaurians as well as tetanurans^36^. The maxilla is also incomplete and with only a general diagnosis possible (e.g. external texture on the maxilla, which is composed of short linear grooves that are also shared with *Majungasaurus* and *Rugops*). The only autapomorphy is a secondary wall in the anteroventral corner of the antorbital fossa obscuring it and that has a scalloped and fluted dorsal margin^36^. Therefore, as the holotype of *Kryptops* is a miscellany of materials belonging to different groups with just one autapomorphy supporting the species, this taxon might have been considered as *nomen dubium* rather than a valid taxon. The postcranial skeleton probably has a phylogenetic relationship with carcharodontosaurids instead of abelisaurids as suggested by Novas *et al*.^4^ and Carrano *et al*.^39^.

Abelisaurids has strongly reduced forearms without grasping ability^40^ (Fig. 3B). According to Agnolin and Chiarelli^40^, abelisaurs probably also lacked forearm mobility. However, recent analyses on *Majungasaurus* musculature suggest that, although much reduced, abelisaurids did not lose full mobility of the forelimb, and may have used it for intraspecific display^41^. Some taxa such as *Aucasaurus*, *Majungasaurus* and *Carnotaurus* may have lost the ungual of the digits I and IV^31,40,42^ whereas the ceratosaurid *Eoabelisaurus* has strongly reduced the manual unguals^12^. The digit IV is fused to the metacarpal in *Majungasaurus* and *Aucasaurus* precluding mobility. Extreme reduction also reduced autonomy of all digits due to the extreme reduction, although the hemispherical humeral head and distal radius and ulna suggests that the shoulder and the wrist had a large range of motion^38,41^. However, as pointed by Gianechini *et al*.^38^ the range of motion of the humerus should have been higher in lateromedially (i.e. abduction-adduction) than in anteroposteriorly (i.e. flexion-extension) because the development of the dorsal and ventral rim of the glenoid fossa reduced anteroposteriorly movements. Also, is worth noting that the large scapulocoracoids and reduced forelimbs in ceratosauroids might be related to a close developmental association between scapular blade and the axial skeleton, holding the shoulder girdle to the axial skeleton and for mobility of the girdle and the ribcage^38,41,43^. Those muscles attached to the neck could have had an important role in feeding as in extant crocodiles (e.g. muscle *levator scapulae* which is an effective abductor of the neck and hence the head)^41,44^.

The hindlimbs of ceratosaurs are different in the two main branches. In noasaurids, the hindlimbs are more slender than the etrigansaurians; however this is due to the overall size of individuals of the groups^1^. Abelisaurids’ hindlimbs and caudal vertebrae suggest that these taxa, specially the brachyrostrans, may have had powerful cursorial abilities. The tibia have well developed dorsal anterior projection (cnemial crest) onto which the main knee extensor muscles are inserted (i.e. *iliotibiales*)^45^. The large size of the cnemial crest and its dorsal inclination suggest that some ankle extensors and digital flexors muscles were large, increasing their force-producing capability. Additionally, the dorsal inclination of the transverse processes in the caudal vertebrae suggests that the muscle *caudofemoralis longus*, the main femur extensor, may have been larger than in other theropods contributing to the cursorial ability^10^. Also, the presence of accessory articulations in caudal vertebrae (hyposphene-hypantrum) apart of the inclined transverse processes, increases the tail rigidity^10,46^ and may have enhanced overall speed and acceleration^10^. However, acceleration might have been more impressive than top speed. When preserved, feet of some abelisaurids are short (e.g. *Majungasaurus*^47^), indicating low tangential velocity at the ankle. The type of *Carnotaurus* lacks feet and the distal portion of the epipodials, even though it is often reconstructed as having gracile legs and feet^17^.

#### Etrigansaurian soft tissue

The etrigansaurians also are well known by their rugosities and projections from the skull elements^3^. Carcharodontosaurid theropods have rugosities in lateral skull bones as well, but the morphology is different^48^ and leads to misinterpretations of the group^49^. Although abelisaurids have strong rugose skulls, the textures are variable throughout the skull^48^. The texturization of the skull happened independently from the projections. For example, the skull of *Ceratosaurus* is diagnosed by having a rounded midline horn core on the fused nasals^3^ and horn cores forming a dorsal crest on the lacrimals^50^, although the skull is otherwise smooth^48^. On the other hand, the skull of *Skorpiovenator bustingorryi* is strongly texturized but without any projections^22^. The skull roof in abelisaurids is thick but this feature varies among the species^48^. Both majungasaurini *Majungasaurus* and *Rajasaurus normandensis* have a single medial horn formed by the frontal and frontal/nasal, respectively^28,48^, whereas the brachyrostran *Carnotaurus* has two frontal horns laterally oriented^17^, *Aucasaurus* has the lateral margins of frontal elevated in the orbital region, and *Viavenator exxoni* has almost flattened frontals^51^. The flattened frontals of *Ekrixinatosaurus novasi*^52^ and probably of *Skorpiovenator* suggest the basal position of these two taxa in relation to Furileusaura as proposed by Filippi *et al*.^15^.

**Figure 4:**
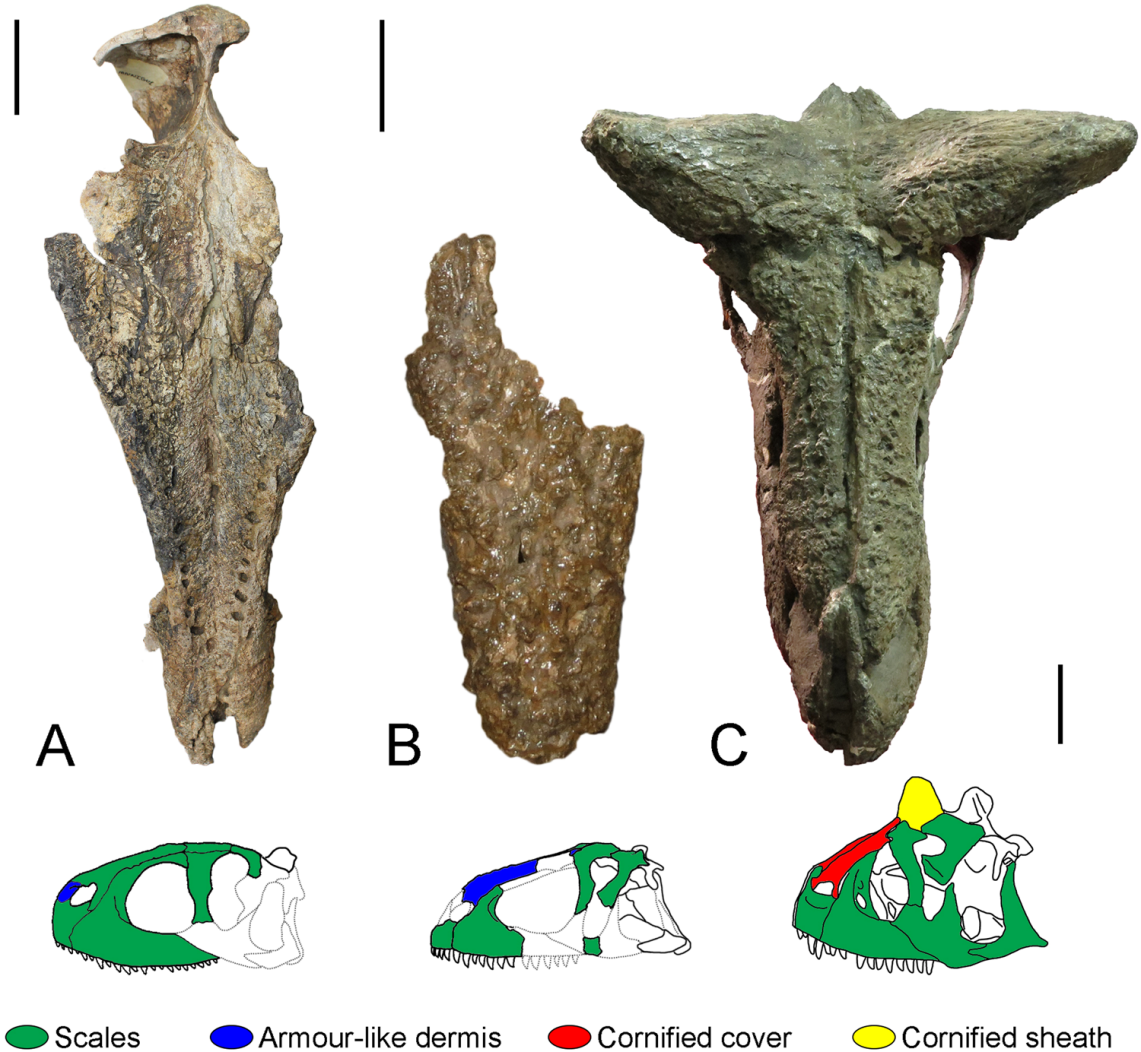
Skin structures inferred for abelisaurids. Dorsal surface of the skull of (**A**) *Rugops* (MNN IGU1), (**C**) *Carnotaurus* (MACN-CH 894) and dorsal surface of the fused nasal of (**B**) *Abelisaurus* (MPCA 11908). Scales bar: 5 cm.

**Figure 5:**
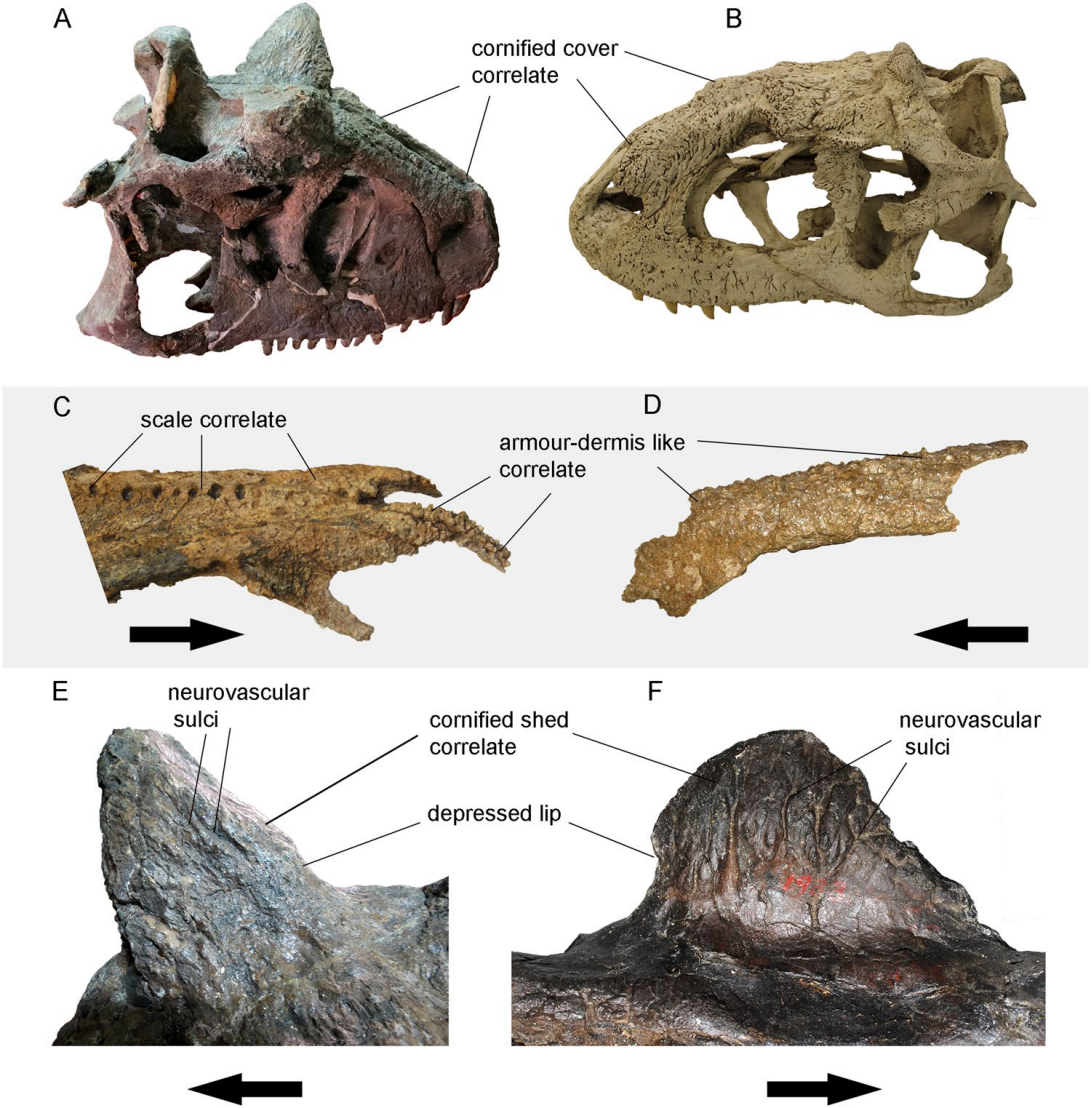
Details of the skin structures inferred for abelisaurids. Right side of the skull of (**A**) *Carnotaurus* (MACN-CH 894) and left side of the skull of (**B**) *Majungasaurus* (FMNH PR 2100 – cast), both in dorsolateral view. Right side of the nasal of (**C**) *Rugops* (MNN IGU1) and left side of the nasal of *Abelisaurus* (MPCA 11908), both in dorsolateral view. Detail of the right frontal horn of (**E**) *Carnotaurus* (MACN-CH 894) and left side of nasal horn of (**F**) *Ceratosaurus* (USNM 4735). Arrowhead pointing rostrally without scale.

The rugosities in abelisaurids resulted from a mineralization processes with specializations in the overlying dermis, such that the mineralized tissue includes the irregular surface texture representing mineralization of the bone’s periosteum, overlying dermal fibers or combination of the two, characterizing the metaplastic ossification^48^. The sculpture of lateral bones (e.g. maxilla, jugal, quadratojugal, dentary) presents a higher percentage of tangential vascular canals and grooves, whereas the dorsal roofing elements (e.g. frontal, dorsal postorbital and lacrimal, nasal, nasal process of the premaxilla) tend to have more projecting, tuberculate and/or cauliflower-like texture that combine with the vascular canals and grooves (Figs 4 and 5A,B)^48^. Sampson and Witmer^48^ have suggested that abelisaurids might have had more robust skulls than other theropods due to the high skull’s mineralization. Following the results of Hieronymus *et al*.^53^ for inference of soft tissues in Centrosaurine and Carr *et al*.^54^ for Tyrannosauridae, it is possible to assess the superficial cranial soft tissues of abelisaurids. These tissues show a hierarchy of textures which became more complex towards the phylogeny.

The basal abelisaurid *Rugops* has the dorsal surface of nasals with a row of seven pits, visible sutures between then and hummocky rugose surface which is also present in the dorsal surface of frontal, prefrontal lacrimal and maxilla (Figs 4A and 5C). These features are correlated with overlying scales as observed in living crocodiles and reptiles^53^. On the other hand, the anterior-most snout has a different texture compared to other categories of soft tissue. The nasal articulation processes of premaxilla and the anterior processes of nasal, show a papillate texture indicating the presence of armour-like dermis as suggested by Hieronymus *et al*.^53^. The presence of these tissues suggests that *Rugops* had, at least two categories of tissues covering the surface of the skull. Interestingly, the type of *Rugops* could be a subadult individual due to its small size, incomplete fusion between the nasals and the presence of the fenestra between the prefrontal, frontal, postorbital and lacrimal^3^. As the rugosities tend to increase during ontogeny^18^, the armour-like dermis could reach a larger surface if *Rugops* grew up and developed more papillate texture.

*Abelisaurus*, as other abelisaurids, have a lateral cranium surface (e.g. maxilla) with dense tangentially arranged grooves suggesting it was covered by large scales or scutes, as suggested by Sampson and Witmer^48^ and Hieronymus *et al*.^53^ (Figs 4B and 5D). However, the nasal of *Abelisaurus* differs from that of *Rugops* being extremely rugose with bones lobules across its surface. This texture is associated with armour-like dermis^53^, as seen in the anterior snout of *Rugops* (Fig. 5C)

The dorsal surface of carnotaurine skulls (nasal, frontal, dorsal lacrimal and dorsal postorbital) have coarse pitting and grooving on bone surfaces suggesting that these were covered by cornified tissue, being an osteological correlate with the cornified cover seen on muskoxen, centrosaurine dinosaurs^53^, and tyrannosaurids^54^ (Figs 4C and 5A,B). However, it is improbable that abelisaurids had projections higher than the frontal horns. This category of tissue increased the toughness of the head roof, which also might have had an important ecological function as discussed below.

The horns of *Carnotaurus* and *Ceratosaurus* would have been more extended than the preserved fossil and covered with cornified sheath, indicated by neurovascular grooves, depressed lip and less rugosity than the other bones surfaces as suggested by the results of Hieronymus *et al*.^53^ (Fig. 5E and F). Although the horn cores of *Carnotaurus* are more rugose than those of *Ceratosaurus*, ventral to the depressed lip the frontals are markedly lesser rugose. The single horn of *Majungasaurus* and *Rajasaurus* do not have the depressed lip seen in *Carnotaurus* and *Ceratosaurus*, suggesting that they were covered by cornified tissue without dorsal extension.

The only preserved soft tissues so far belongs to *Carnotaurus* and correspond to the anterior cervical region associated with cervical ribs, the shoulder region, thorax and tail^17^. The skin impressions present conical protuberances and there is no evidence for filaments or feathers (Fig. 3E). So far, the tubular filaments and feathers are only known in tetanuran theropods^55,56^.

Regarding the bone histology, some analyses also shed some light to the development of ceratosaurs as well as palaeoenvironment^14,57–59^. For example, the robustness of *Masiakasaurus*, once believed as different morphs (robust and gracile)^60^, might be considered to be developmental feature instead of dimorphism^57^, as also shown in allometric analyses^1^. Additionally, the slow growth of the same species can be related to the low resources of Maevarano Formation^57,61^.

### Ceratosaur ontogeny

Ontogenetic traits are difficult to interpret in fossils, sometimes leading to misunderstanding in taxonomy^3,62,63^. In the case of abelisaurs, just a few species are known from certain ontogenetic series, such as *Limusaurus*, *Ceratosaurus* and *Majungasaurus*^3,14,18^. Also, there are some specimens of *Masiakasaurus* of different sizes^33^ with inference on ontogeny from bone histological analyses^57^.

The ontogenetic series of *Ceratosaurus* is still unclear. Madsen and Welles^50^ described two different species of *Ceratosaurus* (*C. magnicornis* and *C. dentisulcatus*) based on cranial and post-cranial associated elements. Nevertheless, Rauhut^64^ suggests that the diagnosis of these species are subjective and there might have been just one species of *Ceratosaurus* in Morrison Formation. Carrano and Sampson^3^, following Rauhut^64^, also argued that these two species have size-based diagnosis suggesting that they might be different ontogenetic specimens from *Ceratosaurus*. Although there are other materials attributed to *Ceratosaurus*^3^, no study was conducted to discuss the ontogenetic traits so far.

The series of *Limusaurus* shows at least 78 ontogenetic modifications through the growth from the analyses of 19 specimens^14^. Delcourt^30^ reported the loss of teeth in mature individuals, while most juveniles had toothed jaws, the skull also becomes longer through ontogeny. In a parallel and broader study, Wang *et al*.^14^ also reported several changes including the formation of a beak after birth. The amount of modifications in *Limusaurus* ontogeny and the presence of gastroliths in the abdominal region also suggest that this species change ontogenetically dietary preferences from omnivory to herbivory^14,32^.

The ontogeny of *Majungasaurus* was assessed by Ratsimbaholison *et al*.^18^ using mainly landmark-based approaches in the skull and in some isolated cranial elements (premaxilla, maxilla, lacrimal, postorbital, jugal, quadrate, dentary and surangular). The authors suggested that the ontogenetic changes include: the skull becomes deeper, the orbit becomes smaller, the sutures among the bones become more complex, and the texture of lateral bones increase^18^. In this study, the postcranial elements were not assessed.

Histological analyses suggest that *Masiakasaurus*^57^ and small abelisaurid theropods^58^ had a cyclical growth strategy as well as slowdown growing. However, in larger taxa, such as *Aucasaurus*, the growth rate tend to be higher than in smaller forms^58^.

Apart from these studies, some inferences about ontogenetic stages were made based on fusion of bones. For example the types of *Xenotarsosaurus bonapartei*, *Eoabelisaurus*, and *Aucasaurus* are considered mature individuals because they have a fused tibia and astragalus^12,42^ (Fig. 3C and D), whereas the type of *Rugops* has been suggested as being an immature individua based on the fusion of the cranial elements^3^ (see above). The type of *Pycnonemosaurus nevesi*, despite being considered the largest abelisaurid so far^1^, is considered a subadult specimen^13^ based on the presence of caudal vertebrae with unfused arches and centra as well as tibia. However, determining the maturity of a specimen based only on the fusion of arches with centrum is not safe because these elements are size-independent^65^.

### Ceratosaur behaviour

Ceratosaur behaviour can be inferred from several studies on anatomy^4,40,48^ and biomechanics^8,9,66^. Also, the new information on soft tissue presented here (see above), suggest a behavioural pattern in abelisaurids as discussed below.

Gregarious behaviour is difficult to deduce; however small species found associated in the same assemblage localities, such as *Masiakasaurus*^33^ and *Limusaurus*^14^, suggest that they might have lived together. In the case of *Majungasaurus*, several specimens were found associated, but some materials (ribs, chevron, neural spines, transverse processes and neural arches) have teeth marks made by its conspecifics suggesting that this species had cannibalistic behaviour^61^. This behaviour can be explained by the resource scarcity in the Maevarano Formation during the Late Cretaceous that was semi-arid^61^.

Going through the new information of soft tissues of abelisaurids shown here (above), it is possible to infer that this clade might have had some intraspecific headbutting matches behaviour at least in carnotaurine taxa (as suggested for *Carnotaurus*^8^ and *Majungasaurus*^67^). The presence of cornified cover on the skull, that was inferred for *Carnotaurus* and *Majungasaurus*, has been related to headbutting behaviour in extant taxa (e.g. *Ovibos moschatus*, *Syncerus caffer* and *Buceros vigil*) as well as extinct (e.g. *Pachyrhinosaurus, Achelousaurus horneri*^53^ and *Stegoceras validum*^68^). Nevertheless, differing from those that engage in violent headbutting and have deep cancellous bone^68^ (which carnotaurine lack), the carnotaurine might have used the head in low-motion headbutting and shoving matches at low speeds (as marine iguana *Amblyrhynchus cristatus*^69^) or engaged giraffe-like strikes to each other’s neck and flanks^67^. The giraffe-like strikes have been proposed for *Majungasaurus*^67^ due to the presence of tall, rugose nasals, struts within sinuses and a unicorn-like projection of the frontals^48,67^, although stresses. Also, the mechanical analyses of *Carnotaurus* skull performed by Mazzetta *et al*.^9^ support the low-motion headbutting in this taxa. Furthermore, the presence of well-developed occipital region (e.g. nuchal crest)^48^ associated with large epipophysis and neural spines in the cervical vertebra increasing the neck musculature^70,71^ strongly suggest that the cervicocephalic complex (head and neck) withstood high stress. Indeed, the well-developed epipophyses indicate a good leverage for intervertebral dorsiflexion by the muscle *tranversospinalis cervicis* and the origin of a strong muscle *complexus*, a head dorsoflexior^72^. As similar features on neck and skull are spread throughout the carnotaurine abelisaurs, all the taxa belonging to this clade may have had similar behaviour in territoriality or mating matches for instance. It is worth noting that cranio-facial biting was reported for non-avian theropods^73–75^. This behaviour could have had several possible reasons, including territoriality, courtship/mating, play, predation/cannibalism, intrapack dominance and subadult dispersal^74^. In the case of carnotaurine, the headbutting and/or giraffe-like strikes could also have been added to the behavioural repertoire for any reasons above.

The low-motion headbutting behaviour also may have been present or began in more basal taxa such as *Rugops* and *Abelisaurus* in parallel with the development of scales and armour-like dermis on the dorsal cranium (e.g. nasal). For example, the dorsal surface of marine iguana skull has hummocky rugosities^53^ as in *Rugops*, suggesting that this structure associated with armour-like dermis might have allowed the abelisaurid a similar behaviour (i.e. low-motion headbutting).This hypothesis of low-motion headbutting developing through the phylogeny in abelisaurids can be tested if a species with similar skull showed *Rugops* hummocky rugosities plus well-developed cervical epipophyses and neural spine and if it was found in Early Cretaceous beds (e.g. Aptian). If the headbutting was not developed in this taxon, certainly the development of armour-like dermis and later cornified cover on the skull in more derived abelisaurids might have allowed for this behaviour. It is worth noting that the giraffe-like strikes seem to be more complex than the iguana-like low-motion headbutting because the first requires more complex development of the skull, as seen in *Majungasaurus*^67^, than in *Rugops*. Therefore, carnotaurine could potentially have adopted both combat styles. The possibilities of these behaviours in abelisaurids are testable with quantitative biomechanical methods^8,9,67^ and could be assessed in the future.

Biomechanical studies on the skull of abelisaurids have suggested that they had cranial mechanical advantage similar to allosaurs (e.g. *Allosaurus fragilis* and *Carcharodontosaurus saharicus*)^66^ and similar bite force (e.g. *Carnotaurus*: 3,341 Newtons^9^; *Allosaurus*: 3,573 Newtons^76^). These results mean that these two groups had high efficient mechanical advantage, but a bite force not as strong as that of *Tyrannosaurus*^9,66^.

According to the analyses of Therrien *et al*.^77^, carnotaurines (e.g. *Majungasaurus* and *Carnotaurus*) might have been ambush predators attacking large prey. Additionally, Sampson and Witmer^48^ have suggested that *Majungasaurus*, and possibly other carnotaurines, were “adapted for a mode of predation that entailed relatively few, penetrating bites accompanied by powerful neck retraction, as well as bite-and-hold behaviour”. This predatory behaviour is consistent with results on skull biomechanics^9,66^ as well as neck analyses^69,70^.

The development of advantageous features (e.g. large muscles for cursorial abilities)^10^ plus the increase the body size towards the phylogeny^1^ granted abelisaurids the opportunity to succeed the carcharodontosaurids as main predators in the Southern Hemisphere after their extinction in Turonian^49,78^. Interestingly, these two groups share dentary^22,49^ and skull advantage mechanics^66^ that might have helped the extinction of carcharodontosaurids through ecological interactions^1^ when this group was becoming rare in the Cenomanian, possibly due to climate changes (i.e. changing in the mean temperatures and floral compositions)^79^. Therefore, it is reasonable to suggest that the latest abelisaurids (carnotaurine) were tyrannoasaurid counterparts since the former were dominant in Southern Hemisphere^3^ and the latter in Northern Hemisphere^2^.

### Ceratosaur biogeography

The new phylogenetic analyses presented by Wang *et al*.^14^ suggest that Ceratosauroidea was present in North America (*Ceratosaurus*) and Asia (*Limusaurus*, also suggested by Rauhut and Carrano^24^), instead just in South America, Europe, Africa, India and Madagascar^4,5^. However, Ceratosauroidea originated in Africa^29^ and the taxonomic diversity spread during the Middle Jurassic to North America, Europe, Asia, Africa, South America and Madagascar (Fig. 1). Australia and Antarctica do not have ceratosaur remains so far^4^, nevertheless it is possible that this group was present there and future discoveries can change this scenario.

The division of the mains branches of Ceratosauroidea (Noasauridae and Etrigansauria) happened in the Early Jurassic^14,29^ just after the origin of this group. The latest ceratosaurs, from the Aptian^36^, were restricted to Southern Hemisphere and Europe^5^. However, during the Barremian to Santonian Gondwana remained isolated from Laurasia when the fauna could acquire a wide geographic distribution across the southern landmass; relating to Europe in Campanian-Maastrichtian rather than Asiamerica^4,80^. The presence of the European majungasaurini *Arcovenator escotae* corroborates this biogeographic hypothesis^5^ whereas the European noasaurid *Genusaurus sisteronis* from Aptian^14,81^ would have to be considered a relic from the early origin of noasaurids.

**Figure 6:**
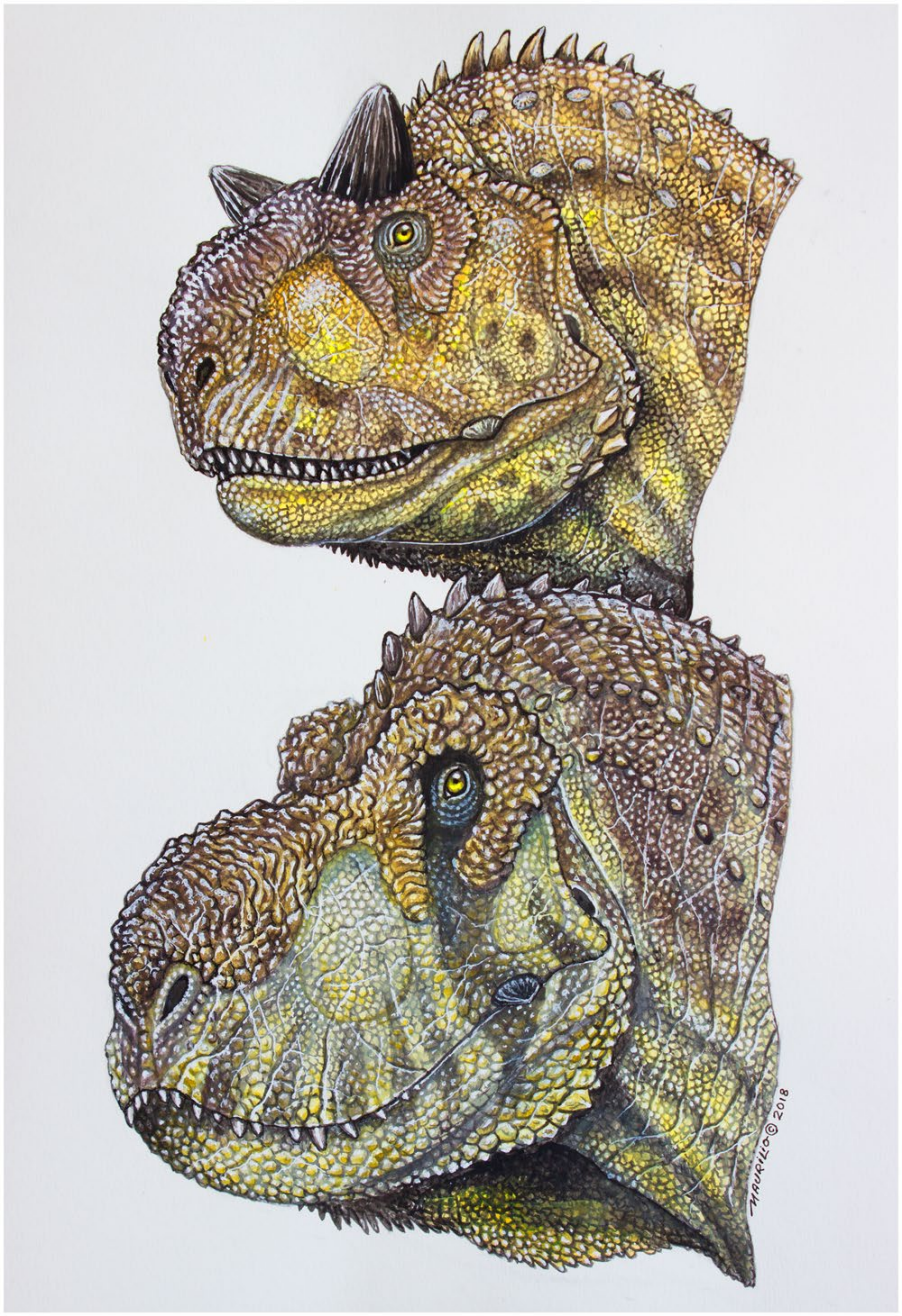
Hypothetical reconstruction of two abelisaurids showing the soft tissues on the head inferred from osteological morphology of the skull. On the top, *Carnotaurus*; on the bottom, *Pycnonemosaurus*. Art by Maurilio Oliveira.

It seems the abelisaurids body size increases along the phylogeny^1^; however, the new phylogenetic analyses presented by Wang *et al*.^14^ suggest a large abelisaur (i.e. *Abelisaurus*) in the base of the clade. Also, there is a new evidence that abelisaurids reached medium/large sizes (between 5.6 and 7.6 m long, based on a partial tibia) from Berriasian-Valanginian of South America^82^. Nevertheless, the largest species were restricted to South America and Africa so far^1,23,83^. This is because insular environments, such as Late Cretaceous of Europe^5^ and Madagascar, supports smaller fauna than continental landmass. Finally, the ability to live in semi-arid palaeoenvironment with low resources, such as those of *Majungasaurus* and *Pycnonemosaurus*^61,84^, and the high disparity of the group facilitated the evolutionary success of ceratosaurs during this time (Fig. 6).

## Methods

The information presented here includes several studies on ceratosaurs anatomy, phylogeny and biomechanics (see References). The soft tissues inference made are based on methods and results presented by Carr *et al*. and and Hieronymus *et al*.^48,49^. Additionally, I examined first-hand the materials of *Abelisaurus comahuensis* (MPCA 11098; Museo Provincial ‘Carlos Ameghino’, Cipolletti, Argentina), *Kryptops palaios* (MNN GAD1-1; Musée National du Niger, Niamey, Niger), *Aucasaurus garridoi* (MCF-PVPH-236; Museo Municipal ‘Carmen Fuñes’, Plaza Huincul, Argentina), *Carnotaurus sastrei* (MACN-CH 894; Museo Argentino de Ciencias Naturales ‘Bernardino Rivadavia’, Buenos Aires, Argentina) *Rugops primus* (MNN IGU1), *Ekrixinatosaurus novasi* (MUCPv-294; Museo de Geologia y Paleontologia, Lago Barreales, Argentina), *Skorpiovenator bustingorryi* (MMCH-PV 48; Museo Minicipal Ernesto Bachman, Villa El Chocon, Argentina), *Majungasaurus crenatissimus* (cast; FMNH PR 2100; Field Museum of Natural History, Chicago, USA), *Ceratosaurus nasicornis* (USNM 4735; National Museum of Natural History, Washington, EUA) for morphological comparison to infer the soft tissues.

## Electronic supplementary material

Supplementary Material
