# Author Correction: Ceratosaur palaeobiology: new insights on evolution and ecology of the southern rulers

**DOI:** 10.1038/s41598-019-43725-2

**Published:** 2019-07-05

**Authors:** Rafael Delcourt

**Affiliations:** 1 Universidade Estadual de Campinas (UNICAMP), Instituto de Geociências, Rua Carlos Gomes, 250, 13083-855 Campinas, SP Brazil; 2 Museu Nacional/Universidade Federal do Rio de Janeiro, Departamento de Geologia e Paleontologia, 20940-040 Rio de Janeiro, RJ Brazil; 3 Department of Zoology, Trinity College Dublin, Dublin, 2 Ireland

Correction to: *Scientific Reports* 10.1038/s41598-018-28154-x, published online 27 June 2018.

This Article contains errors.

In the Results and Discussion section, under the subheading ‘Phylogenetic relationships’,

“In some analyses, *Berberosaurus* is considered as a basal ceratosaurian^12,13^, a neoceratosaurian^5^, a basal abelisauroid^29^ or sister-taxa of cornisauria^14^.”

should read:

“In some analyses, *Berberosaurus* is considered as a basal ceratosaurian^12,13^, a neoceratosaurian^5^, a basal abelisauroid^29^ or sister-taxa of etrigansaurian^14^.”

Furthermore, the Acknowledgements section in this Article is incomplete.

“I would like to thank Paul Sereno and Bob Masek (University of Chicago), David Bohaska (National Museum of Natural History), Juan Porfiri and Domênica Santos (Museo de Ciencias Naturales de la Universidad Nacional del Comahue), Peter Makovicky and William Simpson (Field Museum of Natural History), Rubén Barbieri (Museo Provincial Carlos Ameghino), Rubén Martínez (Universidad Nacional de la Patagonia San Juan Bosco), Rodolfo Coria (Museo Municipal Carmen Fuñes), Juan Canale (Museo Municipal Ernesto Bachmann), Alejandro Kramarz and Federico Agnolín (Museo Argentino de Ciencias Naturales ‘Bernardino Rivadavia’), Eduardo Ruigómez (Museo Paleontológico ‘Egidio Feruglio’) and Xu Xing and Zheng Fang (Institute of Vertebrate Paleontology and Paleoanthropology) for providing access to their respective palaeontological collections. I thank Maurilio Oliveira for providing the Fig. 6. I also thank Ulisses Caramaschi and Borja Holgado (Museu Nacional) for important comments on nomenclature and phylogenetic definitions. I thank Gwendoline Deslyper (Trinity College Dublin) and Nadine Douglas (University College Dublin) for reviewing the English language. Also, I thank Fundação de Amparo à Pesquisa do Estado de São Paulo and Coordenação de Aperfeiçoamento de Pessoal de Nível Superior for their financial support (FAPESP 2012/09370-2; CAPES - PNPD).”

should read:

“I would like to thank Paul Sereno and Bob Masek (University of Chicago), David Bohaska (National Museum of Natural History), Juan Porfiri and Domênica Santos (Museo de Ciencias Naturales de la Universidad Nacional del Comahue), Peter Makovicky and William Simpson (Field Museum of Natural History), Rubén Barbieri (Museo Provincial Carlos Ameghino), Rubén Martínez (Universidad Nacional de la Patagonia San Juan Bosco), Rodolfo Coria (Museo Municipal Carmen Fuñes), Juan Canale (Museo Municipal Ernesto Bachmann), Alejandro Kramarz and Federico Agnolín (Museo Argentino de Ciencias Naturales ‘Bernardino Rivadavia’), Eduardo Ruigómez (Museo Paleontológico ‘Egidio Feruglio’) and Xu Xing and Zheng Fang (Institute of Vertebrate Paleontology and Paleoanthropology) for providing access to their respective palaeontological collections. I thank Maurilio Oliveira for providing the Fig. 6. I also thank Ulisses Caramaschi and Borja Holgado (Museu Nacional) for important comments on nomenclature and phylogenetic definitions. I thank Gwendoline Deslyper (Trinity College Dublin) and Nadine Douglas (University College Dublin) for reviewing the English language. I would like to thank Hussam El Dine Zaher (Museu de Zoologia da Universidade de São Paulo) for providing me supervision during my Ph.D. studies and to Museu de Zoologia da Universidade de São Paulo itself that hosted me for 4 wonderful years. Many thanks to all its employers. Also, I thank Fundação de Amparo à Pesquisa do Estado de São Paulo and Coordenação de Aperfeiçoamento de Pessoal de Nível Superior for their financial support (FAPESP 2012/09370-2; CAPES - PNPD).”

