# Supplementary material for: Ceratosaur palaeobiology: new insights on evolution and ecology of the southern rulers
Source: Sci Rep. 2018 Jun 27;8:9730. doi: 10.1038/s41598-018-28154-x (PMC6021374; doi:10.1038/s41598-018-28154-x)
Supplement: Supplementary file 1 — Supplementary Material [file 41598_2018_28154_MOESM1_ESM.docx]

Supplementary material for **Ceratosaur palaeobiology: new insights on evolution and ecology of the southern rulers**

Rafael Delcourt

Universidade Estadual de Campinas (UNICAMP), Instituto de Geociências, Rua Carlos Gomes, 250, 13083-855 Campinas, SP, Brazil

Museu Nacional/Universidade Federal do Rio de Janeiro, Departamento de Geologia e Paleontologia, 20940-040, Rio de Janeiro, RJ, Brazil

Department of Zoology, Trinity College Dublin, Dublin 2, Ireland. [rafael.delcourt@gmail.com](mailto:rafael.delcourt@gmail.com)

1 – Supplementary Table S1. Ceratosauroidea taxa and its original description.

2 – Phylogenetic definitions.

1 - Supplementary Table S1. Ceratosauroidea taxa and its original description.

| Taxon | Systematic | Location | Age | Reference |
| --- | --- | --- | --- | --- |
| *Berberosaurus liassicus* | Ceratosauroidea | Morocco | Pliensbachian–Toarcian | Allain et al.^1^ |
| *Ceratosaurus nasicornis* | Ceratosauridae | EUA | Kimmeridgian–Tithonian | Marsh^2^ |
| *Eoabelisaurus mefi* | Ceratosauridae | Argentina | Aalenian-Bajocian | Pol and Rauhut^3^ |
| *Genyodectes serus* | Ceratosauridae | Argentina | Aptian–Albian | Woodward^4^ |
| *Rugops primus* | Abelisauridae | Niger | Cenomanian | Sereno et al.^5^ |
| *Abelisaurus comahuensis* | Abelisauridae | Argentina | early–middle Campanian | Bonaparte and Novas^6^ |
| *Ilokelesia aguadagrandensis* | Abelisauridae | Argentina | Late Cenomanian–Early Turonian | Coria and Salgado^7^ |
| *Aucasaurus garridoi* | Furileusauria | Argentina | early–middle Campanian | Coria et al.^8^ |
| *Carnotaurus sastrei* | Furileusauria | Argentina | Maastrichtian | Bonaparte^9^ |
| *Skorpiovenator bustingorryi* | Brachyrostra | Argentina | Late Cenomanian–Early Turonian | Canale et al.^10^ |
| *Quilmesaurus curriei* | Furileusauria | Argentina | Late Campanian–early Maastrichtian | Coria^11^ |
| *Ekrixinatosaurus novasi* | Brachyrostra | Argentina | late? Cenomanian | Calvo et al.^12^ |
| *Pycnonemosaurus nevesi* | Furileusauria | Brazil | Campanian-Masstrichitian | Kellner and Campos^13^ |
| *Viavenator exxoni* | Furileusauria | Argentina | Santonian | Filippi et al.^14^ |
| *Chenanisaurus barbaricus* | Etrigansauria | Morocco | Late Maastrichtian | Longrich et al.^15^ |
| *Indosaurus matleyi* | Majungasaurinae | India | Maastrichtian | Huene and Matley^16^ |
| *Arcovenator escotae* | Majungasaurini | France | Late Campanian | Tortosa et al.^17^ |
| *Dahalokely tokana* | Majungasaurini | Madagascar | Turonian | Farke and Sertich^18^ |
| *Majungasaurus crenatissimus* | Majungasaurini | Madagascar | ?upper Campanian–Maastrichtian | Depéret (Lavocat)^19,20^ |
| *Rajasaurus narmadensis* | Majungasaurini | India | Maastrichtian | Wilson et al.^21^ |
| *Rahiolisaurus gujaratensis* | Majungasaurini | India | Maastrichtian | Novas et al.^22^ |
| *Spinostropheus gautieri* | Noasauridae | Niger | Neocomian | Sereno et al.^5^ |
| *Ligabueino andesi* | Noasauridae | Argentina | Barremian–early Aptian | Bonaparte^23^ |
| *Elaphrosaurus bambergi* | Elaphrosaurinae | Tanzania | Kimmeridgian–Tithonian | Janensh^24^ |
| *Limusaurus inextricabilis* | Elaphrosaurinae | China | Oxfordian | Xu et al.^25^ |
| *Deltadromeus agilis* | Noasaurinae | Morocco | early Cenomanian | Sereno et al.^26^ |
| *Laevisuchus indicus* | Noasaurinae | India | Maastrichtian | Huene and Matley^16^ |
| *Genusaurus sisteronis* | Noasaurinae | France | Albian | Accarie et al.^27^ |
| *Noasaurus leali* | Noasaurinae | Argentina | ?upper Campanian–Maastrichtian | Bonaparte and Powell^28^ |
| *Velocisaurus unicus* | Noasaurinae | Argentina | Coniacian | Bonaparte^29^ |
| *Masiakasaurus knoplferi* | Noasaurinae | Madagascar | ?upper Campanian–Maastrichtian | Sampson et al.^30^ |
| *Austrocheirus isasii* | Noasaurinae | Argentina | early Maastrichtian | Ezcurra et al.^31^ |

2 – Phylogenetic definitions.

Several higher-level theropod taxa used here are defined as follows from different sources as well as new definitions.

**Ceratosauria**: Most inclusive clade containing *Ceratosaurus* but not *Neornithes*^32^

**Ceratosauroidea**: *Carnotaurus*, *Noasaurus* and all their most recent common ancestors and all descendants^21^

**Noasauridae**: Most inclusive clade containing *Noasaurus* but not *Carnotaurus*^21^

**Elaphrosaurinae**: Most inclusive clade containing *Elaphrosaurus* but not *Noasaurus*^33^

**Noasaurinae**: Most inclusive clade containing *Noasaurus* but not *Elaphrosaurus*^33^

**Etrigansauria**: (new clade) the most inclusive clade containing *Carnotaurus* and *Ceratosaurus* but not *Noasaurus*. Etrigansauria means “daemon lizard Etrigan”, a daemon from DC Comics mythology.

**Ceratosauridae**: (new definition) the most inclusive clade containing *Ceratosaurus* but not *Carnotaurus*.

**Abelisauridae**: (new definition) the most inclusive clade containing *Carnotaurus* but not *Ceratosaurus*.

**Carnotaurinae**: Most inclusive clade containing *Carnotaurus* but not *Abelisaurus*^34^

**Majungasaurini**: Most inclusive clade containing *Majungasaurus* but not *Carnotaurus*^17^

**Brachyrostra**: Most inclusive clade containing *Carnotaurus* but not *Majungasaurus*^10^

**Furileusauria**: Most inclusive clade containing *Carnotaurus* but not *Skorpiovenator*^14^

Supplementary references

1. Allain, R. *et al.* An abelisauroid (Dinosauria: Theropoda) from the Early Jurassic of the High Atlas Mountains, Morocco, and the radiation of ceratosaurs. *Journal of Vertebrate Paleontology* **27,** 610–624 (2007).

2. Marsh, O. C. Principal characters of American Jurassic dinosaurs, Part VIII, The Order Theropoda. *Am. J. Sci.* **27,** 329–340 (1884).

3. Pol, D. & Rauhut, O. W. M. A Middle Jurassic abelisaurid from Patagonia and the early diversification of theropod dinosaurs. *Proc. R. Soc. B Biol. Sci.* **279,** 1–6 (2012).

4. Woodward, A. S. On some extinct reptiles from Patagonia, of the genera Miolania, Dinilysia, and Genyodectes. *Proc. Zool. Soc. London* **1901,** 169–184 (1901).

5. Sereno, P. C., Wilson, J. a & Conrad, J. L. New dinosaurs link southern landmasses in the Mid-Cretaceous. *Proc. Biol. Sci.* **271,** 1325–1330 (2004).

6. Bonaparte, J. & Novas, F. Abelisaurus comahuensis n. gen. n. sp. Carnosauria del cretácico superior de Patagonia. *Ameghiniana* **21,** 259–265 (1985).

7. Coria, R. A. & Salgado, L. A basal Abelisauria Novas, 1992 (Theropoda: Ceratosauria) from the Cretaceous of Patagonia, Argentina. *Gaia Ecol. Perspect. Sci. Soc.* **102,** 89–102 (1998).

8. Coria, R. A., Chiappe, L. M. & Dingus, L. A new close relative of *Carnotaurus sastrei* Bonaparte 1985 (Theropoda: Abelisauridae) from the Late Cretaceous of Patagonia. *J. Vertebr. Paleontol.* **22,** 460–465 (2002).

9. Bonaparte, J. F. A horned Cretaceous carnosaur from Patagonia. *National Geographic Research* **1,** 149–151 (1985).

10. Canale, J. I., Scanferla, C. a, Agnolin, F. L. & Novas, F. E. New carnivorous dinosaur from the Late Cretaceous of NW Patagonia and the evolution of abelisaurid theropods. *Naturwissenschaften* **96,** 409–14 (2009).

11. Coria, R. A. in *Mesozoic vertebrate life* (eds. Tanke, D. H. & Carpenter, K.) **1,** 1–7 (Indiana University Press Bloomington, 2001).

12. Calvo, J. O., Rubilar-Rogers, D. & Moreno, K. A new Abelisauridae (Dinosauria: Theropoda) from northwest Patagonia. *Ameghiniana* **41,** 555–563 (2004).

13. Kellner, A. A. & Campos, D. A. On a new theropod dinosaur (Abelisauria) from the continental Cretaceous of Brazil. *Arq. do Mus. Nac.* **60,** 163–170 (2002).

14. Filippi, L. S., Méndez, A. H., Juárez Valieri, R. D. & Garrido, A. C. A new brachyrostran with hypertrophied axial structures reveals an unexpected radiation of latest Cretaceous abelisaurids. *Cretaceous Research* **61,** 209–219 (2016).

15. Longrich, N. R., Pereda-Suberbiola, X., Jalil, N.-E., Khaldoune, F. & Jourani, E. An abelisaurid from the latest Cretaceous (late Maastrichtian) of Morocco, North Africa. *Cretac. Res.* **76,** 40–52 (2017).

16. Huene, F. R. & Matley, C. A. *The Cretaceous Saurischia and Ornithischia of the Central provinces*. (Manager of publications, 1933).

17. Tortosa, T. *et al.* A new abelisaurid dinosaur from the Late Cretaceous of southern France: Palaeobiogeographical implications. *Ann. Paleontol.* **100,** 63–86 (2014).

18. Farke, A. A. & Sertich, J. J. W. An Abelisauroid Theropod Dinosaur from the Turonian of Madagascar. *PLoS ONE* **8,** (2013).

19. Depéret, C. Note sur les dinosauriens sauropodes et théropodes du Crétacé supérieur de Madagascar. *Bull. la Société Géologie Française* **21,** 176–196 (1896).

20. Lavocat, R. Sur une portion de mandibule de théropode provenant du Crétacé supérieur de Madagascar. *Bull. la Museum Natl. d’Histoire Nat. (sér. 2)* **27,** 256–259 (1955).

21. Wilson, J. A. *et al.* A new abelisaurid (Dinosauria, Theropoda) from the Lameta Formation (Cretaceous, Maastrichtian) of India. (2003).

22. Novas, F. E., Chatterjee, S., Rudra, D. K. & Datta, P. M. in *New aspects of Mesozoic biodiversity* (ed. Bandyopadhyay, S.) 45–62 (Springer, 2010).

23. Bonaparte, J. F. Cretaceous tetrapods of Argentina. *Münchner Geowissenschaftliche Abhandlungen* **30,** 73–130 (1996).

24. Janensch, W. Über *Elaphrosaurus bambergi* und die megalosaurier aus den Tendaguru-Schichten Deutsch-Ostafrikas. *Sitzungsberichte der Gesellschaft Naturforschender Freunde zu Berlin* 226–235 (1920).

25. Xu, X. *et al.* A Jurassic ceratosaur from China helps clarify avian digital homologies. *Nature* **459,** 940–4 (2009).

26. Sereno, P. C. *et al.* Predatory dinosaurs from the Sahara and Late Cretaceous Faunal Differentiation. *Science* **272,** 986–991 (1996).

27. Accarie, H. *et al.* Découverte d’un dinosaure théropode nouveau (*Genusaurus sisteronis* ng, n. sp.) dans l’Albien marin de Sisteron (Alpes de Haute-Provence, France) et extension au Crétacé inférieur de la lignée cératosaurienne. *Comptes rendus l’Académie des Sci. Série 2. Sci. la terre des planètes* **320,** 327–334 (1995).

28. Bonaparte, J. F. & Powell, J. E. A continental assemblage of tetrapods from the Upper Cretaceous beds of El Brete, northwestern Argentina (Sauropoda-Coelurosauria-Carnosauria-Aves). *Mem. la Soc. Geol. la Fr. (Nouvelle Ser.* **139,** 19–28 (1980).

29. Bonaparte, J. F. The Gondwanian theropod families Abelisauridae and Noasauridae. *Hist. Biol.* **5,** 1–25 (1991).

30. Sampson, S. D., Carrano, M. T. & Forster, C. A. A bizarre predatory dinosaur from the Late Cretaceous of Madagascar. *Nature* **409,** 504–6 (2001).

31. Ezcurra, M. D., Agnolin, F. L. & Novas, F. E. An abelisauroid dinosaur with a non-atrophied manus from the Late Cretaceous Pari Aike Formation of southern Patagonia. *Zootaxa* **2450,** 1–25 (2010).

32. Padian, K., Hutchinson, J. R. & Holtz, T. R. Phylogenetic definitions and nomenclature of the major taxonomic categories of the carnivorous Dinosauria (Theropoda). *J. Vertebr. Paleontol.* **19,** 69–80 (1999).

33. Rauhut, O. W. M. & Carrano, M. T. The theropod dinosaur *Elaphrosaurus bambergi* Janensch, 1920, from the Late Jurassic of Tendaguru, Tanzania. *Zool. J. Linn. Soc.* (2016). doi:10.1111/zoj.12425

34. Sereno, P. C. A rationale for phylogenetic definitions, with application to the higher-level taxonomy of Dinosauria. *Neues Jahrb. fuer Geol. und Palaeontol. Abhandlungen* **210,** 41–83 (1998).
